# Impact of gender: Rivaroxaban for patients with atrial fibrillation in the XANTUS real‐world prospective study

**DOI:** 10.1002/clc.23452

**Published:** 2020-09-08

**Authors:** A. John Camm, Pierre Amarenco, Sylvia Haas, Miriam Bach, Paulus Kirchhof, Silvia Kuhls, Marc Lambelet, Alexander G.G. Turpie, XANTUS Investigators

**Affiliations:** ^1^ Cardiovascular and Cell Sciences Research Institute, St George's University of London London UK; ^2^ Department of Neurology and Stroke Centre Paris‐Diderot‐Sorbonne University Paris France; ^3^ Formerly Technical University Munich Munich Germany; ^4^ Medical Affairs Bayer AG Berlin Germany; ^5^ Institute of Cardiovascular Sciences, University of Birmingham, University Hospital Birmingham NHS Foundation Trust and Sandwell and West Birmingham Hospitals NHS Trust Birmingham UK; ^6^ Department of Cardiology University Heart and Vascular Center UKE Hamburg Germany; ^7^ Integrated Analysis Statistics, Bayer AG Wuppertal Germany; ^8^ Chrestos Concept GmbH and Co KG Essen Germany; ^9^ Department of Medicine, McMaster University, Hamilton ON Canada

**Keywords:** anticoagulant, atrial fibrillation, real‐world evidence, stroke

## Abstract

**Background:**

The XANTUS study (NCT01606995) demonstrated low rates of stroke and major bleeding in patients with atrial fibrillation (AF) receiving rivaroxaban in clinical practice for the prevention of thromboembolic events (N = 6784).

**Hypothesis:**

Because previous real‐world studies have not reported gender‐dependent responses to rivaroxaban treatment, this sub‐analysis of the XANTUS study investigated the effect of gender on outcomes.

**Methods:**

The centrally adjudicated outcomes were compared between genders. Primary outcomes were major bleeding and all‐cause death. Secondary outcomes included symptomatic thromboembolic events. Multivariable Cox regression analysis was performed to assess the effect of risk factors on outcomes between genders.

**Results:**

A total of 2765 female and 4016 male patients were included in the analysis. Baseline characteristics were generally similar. No nominally significant interaction between gender and risk factors for the study outcomes was observed. Rates of major bleeding, all‐cause death and symptomatic thromboembolic events in patients with non‐valvular AF receiving rivaroxaban for stroke prevention were similar in men and women; no significant differences in risk factors for these outcomes were observed between genders.

**Conclusions:**

Further research is needed to better characterize the relative importance of different risk factors on outcomes in men vs women and to determine whether gender differences exist in patients treated with non‐vitamin K antagonist oral anticoagulants.

## 1 INTRODUCTION

Atrial fibrillation (AF) is a common cardiac arrhythmia.^1^ Its reported prevalence has increased in recent years, due to the aging population and perhaps partly due to improved detection.^1^, ^2^ AF is associated with a 5‐fold increase in the risk of stroke. The risk of AF is higher in men than women, with a ratio of 1.2:1.^3^ According to data from the Framingham Heart Study, the prevalence of AF in men is nearly twice that in women when adjusting for age.^4^ However, because the prevalence of AF increases with age and, on average, women live longer than men, it is estimated that at least as many women as men have AF.^5^, ^6^ In contrast, women with AF have a higher risk of stroke than men with AF, and AF‐related strokes appear to be more severe in women.^7^, ^8^ Female gender is a recognized risk factor in the CHA_2_DS_2_‐VASc stroke risk stratification scheme.^1^, ^7^

XANTUS was an international, prospective, observational study designed to assess the safety and effectiveness of rivaroxaban for stroke prevention in routine clinical practice in patients with nonvalvular AF newly initiated on rivaroxaban (N = 6784).^9^ In this study, patients taking rivaroxaban had low rates of stroke and major bleeding; most patients (96.1%) did not experience a major event (major bleeding, death or stroke/systemic embolism [SE]) during the study.^9^

There is some evidence that gender‐dependent responses to anticoagulant treatment may exist, such as a smaller reduction in stroke/SE risk with anticoagulation in women compared with men.^10^ Because this could have important clinical implications, we performed an analysis of the XANTUS data to investigate the impact of gender on the rates of major bleeding, symptomatic thromboembolic events and death in patients with AF receiving rivaroxaban in clinical practice.

## 2 Methods

The design of the international, noninterventional, observational XANTUS study was approved by the European Medicines Agency. The study design and main results have been published previously.^9^, ^11^ Briefly, men and women (aged ≥18 years) with AF initiated on rivaroxaban therapy for stroke/SE prevention were enrolled. Patients had follow‐up visits at approximately 3month intervals for 1 year; or approximately 30 days after permanent discontinuation of rivaroxaban, if treatment was discontinued before 1 year.

Primary outcomes were chosen to assess the safety of rivaroxaban in clinical practice and consisted of major bleeding events (International Society on Thrombosis and Haemostasis definition^12^) and all‐cause death. Secondary outcomes included symptomatic thromboembolic events: stroke, noncentral nervous system SE (nonCNS SE), transient ischemic attack (TIA) and myocardial infarction. These outcomes were adjudicated by a Central Adjudication Committee.

The analyses were based on the safety population, which included all patients who received at least one dose of rivaroxaban. Only treatment‐emergent events were included in the analyses. An event was considered treatment emergent if it occurred on or after the day of the first dose of rivaroxaban and up to 2 days after the last dose. No imputation of missing data was applied, that is, patients with missing data for the variables required for multivariable Cox regression modeling were not included in the analyses. However, a large proportion of patients (34.4%) had missing creatinine clearance (CrCl) measurements. Because the rates of treatment‐emergent major outcomes were lower in patients with missing CrCl data (compared with patients with available CrCl measurements) and their baseline characteristics were similar to those of patients with CrCl ≥50 mL/minute, these patients were included in the analysis by grouping them with patients with CrCl ≥50 mL/minute.

To identify differences in the influence of risk factors on outcomes between genders, a multivariable Cox regression model was fitted; it included the risk factors of interest as well as gender interactions. Based on this model, the effect of risk factors on outcomes was displayed separately for women and men, with interaction *P* values indicated. Risk factors for each outcome were selected based on medical judgment, and included risk factors in the CHADS_2_, HAS‐BLED, and CHA_2_DS_2_‐VASc scores as well as risk factors identified in previous studies.^10^ This sub‐analysis was of an exploratory, descriptive nature. Hazard ratios (HRs) with 95% confidence intervals (CIs) were calculated before and after adjusting for differences in baseline risk factors to explore directional trends.

## 3 Results

Of the 6784 patients included in the safety population, 4016 (59.2%), were male and 2765 (40.8%) were female. On average, women were older than men (mean 73.7 vs 70.0 years old, respectively; *P* < .0001), and more women were aged >75 years (46.3% vs 30.9%; *P* < .0001) (Table 1). Compared with men, women had higher risk scores for stroke (mean CHADS_2_ score 2.1 vs 1.9 in men; *P* < .0001) and bleeding (mean HAS‐BLED score 2.1 vs 2.0 in men; *P* < .0001), as well as higher rates of hypertension (77.0% vs 73.1% in men; *P* = .0002). The mean CHA_2_DS_2_‐VASc score was 4.1 in women and 2.9 in men (*P* < .0001). However, being female adds one point to this score: after excluding gender, the mean CHA_2_DS_2_‐VASc scores were 3.1 for women and 2.9 for men (*P* < .0001). Because female gender accounted for most of the difference in CHA_2_DS_2_‐VASc score, it is unlikely that other factors such as age > 75 years contributed substantially to the difference in CHA_2_DS_2_‐VASc score between men and women.

**TABLE 1:** Baseline characteristics of patients treated with rivaroxaban in XANTUS with available data relating to gender

Baseline characteristic	Men(n = 4016)	Women(n = 2765)	*P* Value^a^
Age, years, mean ± SD	70.0 ± 10.0	73.7 ± 9.5	<.0001 <.0001
<65 y, n (%)	1051 (26.2)	427 (15.4)	
≥65‐≤ 75 y, n (%)	1723 (42.9)	1058 (38.3)	
>75 y, n (%)	1242 (30.9)	1280 (46.3)	
First available weight, kg, mean ± SD	88.6 ± 16.3	74.8 ± 15.4	<.0001
Body mass index, mean ± SD	28.5 ± 4.6	28.0 ± 5.5	.0004
First available creatinine clearance, n (%)			<.0001
<15 mL/minute	12 (0.3)	8 (0.3)	
15 to < 30 mL/minute	32 (0.8)	43 (1.6)	
≥30 to < 50 mL/minute	232 (5.8)	313 (11.3)	
≥50 to ≤ 80 mL/minute	1331 (33.1)	1022 (37.0)	
>80 mL/minute	1013 (25.2)	444 (16.1)	
Data missing	1396 (34.8)	935 (33.8)	
Existing co‐morbidities, n (%)			
Hypertension	2934 (73.1)	2129 (77.0)	.0002
Diabetes	836 (20.8)	497 (18.0)	.0038
Congestive heart failure	753 (18.8)	512 (18.5)	.8089
Prior stroke/TIA/nonCNS SE	720 (17.9)	571 (20.7)	.0050
Prior myocardial infarction	516 (12.8)	172 (6.2)	<.0001
Vascular disease^b^	1109 (27.6)	576 (20.8)	<.0001
Type of atrial fibrillation, n (%)			<.0001
First diagnosed	728 (18.1)	524 (19.0)	
Paroxysmal	1537 (38.3)	1218 (44.1)	
Persistent	601 (15.0)	322 (11.6)	
Permanent	1144 (28.5)	691 (25.0)	
Data missing	6 (0.1)	10 (0.4)	
CHADS_2_ score, mean ± SD	1.9 (1.3)	2.1 (1.3)	<.0001
CHA_2_DS_2_‐VASc score, mean ± SD	2.9 (1.6)	4.1 (1.6)	<.0001
HAS‐BLED score, mean ± SD	2.0 (1.0)	2.1 (1.0)	<.0001
Prior antithrombotic therapy, n (%)			.0006
Yes	2998 (74.7)	1960 (70.9)	
No	1018 (25.3)	805 (29.1)	
Type of prior antithrombotic therapy, n (%)			.0046
Vitamin K antagonist	1649 (41.1)	1118 (40.4)	
Direct thrombin inhibitor	122 (3.0)	86 (3.1)	
Direct Factor Xa inhibitor	8 (0.2)	2 (0.1)	
Acetylsalicylic acid	744 (18.5)	479 (17.3)	
Dual antiplatelet therapy	51 (1.3)	17 (0.6)	
Heparin	130 (3.2)	87 (3.1)	
Other antithrombotic therapy	36 (0.9)	19 (0.7)	
Multiple	258 (6.4)	152 (5.5)	
Hospitalized at baseline, n (%)			.042
Yes	689 (17.2)	537 (19.4)	
No	3326 (82.8)	2228 (80.6)	
Data missing	1 (< 0.05)	0 (0.0)	

Abbreviations: NonCNS SE, noncentral nervous system systemic embolism; SD, standard deviation; TIA, transient ischemic attack.

^a^ Based on chi‐square test for categorical variables and *t*‐test for continuous variables.

^b^ Defined as peripheral artery disease, ischemic heart disease or cerebrovascular disease.

Some differences in baseline co‐morbidities were apparent between men and women (Table 1): women were more likely to have a history of stroke/TIA/nonCNS SE (20.7% vs 17.9% in men; *P* = .0050), but fewer had diabetes (18.0% of women vs 20.8% of men; *P* = .0038) or a history of myocardial infarction (6.2% of women vs 12.8% of men; *P* < .0001). Similar proportions of women (66.2%) and men (65.2%) had available CrCl measurements (first available CrCl, Table 1). Among patients with available CrCl measurements, more women than men had moderate‐to‐severe renal impairment (CrCl <50 mL/minute; 13.2% of women vs 6.9% of men), qualifying them for a reduced dose of rivaroxaban according to the label recommendation.^13^ A total of 3279 men (81.6%) and 2054 women (74.3%) received rivaroxaban 20 mg once daily, while 717 men (17.9%) and 693 women (25.1%) received a reduced dose of rivaroxaban 15 mg once daily. Less than 1% of patients received other or unknown doses.

Before adjusting for baseline differences, men and women had a similar risk of major bleeding (HR = 1.18 for men vs women; 95% CI 0.82‐1.69; *P* = .368), stroke/nonCNS SE (HR = 0.90; 95% CI 0.52‐1.57; *P* = .714), all‐cause death (HR = 0.87; 95% CI 0.60‐1.25; *P* = .449), the composite of major bleeding, all‐cause death, stroke or nonCNS SE (HR = 0.93; 95% CI 0.73‐1.18; *P* = .539), myocardial infarction (HR = 1.37; 95% CI 0.62‐3.05; *P* = .440) or ischemic stroke (HR = .68; 95% CI 0.34‐1.37; *P* = .284).

**FIGURE 1:**
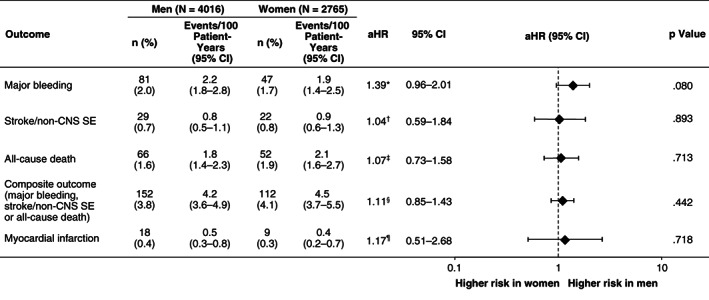
Adjusted outcomes by gender*Adjusted for age, first available CrCl, CHF, uncontrolled hypertension, prior stroke/TIA/nonCNS SE, prior bleeding, vascular disease, liver disease and baseline antiplatelets/acetylsalicylic acid/nonsteroidal anti‐inflammatory drugs; ^†^adjusted for age, first available CrCl, CHF, hypertension, prior stroke/TIA/nonCNS SE, vascular disease and diabetes mellitus; ^‡^adjusted for age, first available CrCl, CHF, hypertension, prior stroke/TIA/nonCNS SE, vascular disease, diabetes mellitus and current smoker; ^§^adjusted for age, first available CrCl, CHF, hypertension, prior bleeding, prior stroke/TIA/nonCNS SE, vascular disease, diabetes mellitus, current smoker, baseline antiplatelets/acetylsalicylic acid/nonsteroidal anti‐inflammatory drugs and liver disease; ^¶^adjusted for age, hypertension, vascular disease, diabetes mellitus and current smoker aHR, adjusted hazard ratio (men vs women); CHF, congestive heart failure; CI, confidence interval; CrCl, creatinine clearance; HR, hazard ratio; nonCNS SE, noncentral nervous system systemic embolism; TIA, transient ischemic attack

After adjustment for differences in baseline risk factors, men and women still had similar risks of all major outcomes (Figure 1). There was a nonsignificant trend towards a higher risk of major bleeding in men compared with women (HR = 1.39; 95% CI 0.96‐2.01; *P* = .080). The risk of stroke/nonCNS SE (HR 1.04; 95% CI 0.59‐1.84; *P* = .893) and all‐cause death (HR = 1.07; 95% CI 0.73‐1.58; *P* = .713) were similar between genders. Finally, there were no nominally significant differences between genders in the risk of the composite of major bleeding, all‐cause death, stroke or nonCNS SE (HR = 1.11; 95% CI 0.85‐1.43; *P* = .442) or the individual outcomes of myocardial infarction (HR = 1.17; 95% CI 0.51‐2.68; *P* = .718) or ischemic stroke (HR = 0.79; 95% CI 0.39‐1.61; *P* = .518).

**FIGURE 2:**
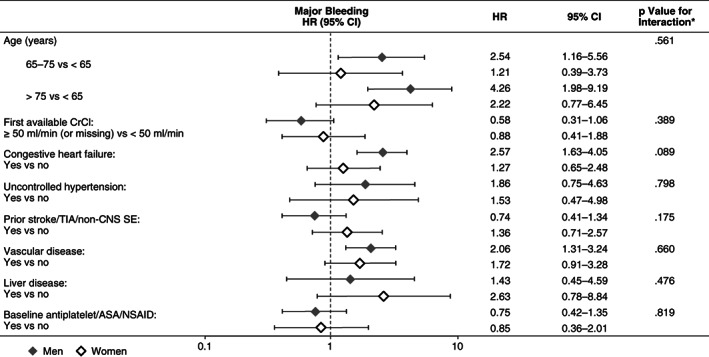
Influence of risk factors on major bleeding by gender *P value is based on the interaction between gender and the individual risk factor. ASA, acetylsalicylic acid; CI, confidence interval; CrCl, creatinine clearance; HR, hazard ratio; nonCNS SE, noncentral nervous system systemic embolism; NSAID, nonsteroidal anti‐inflammatory drug; TIA, transient ischemic attack

Analysis of the influence of different risk factors on major bleeding, stroke/SE, all cause‐death and the composite outcome revealed broadly consistent results between genders. Several nonsignificant trends were observed. Advancing age and congestive heart failure (CHF) tended to be associated with a greater increase in the risk of major bleeding in men than in women, while a history of vascular disease was associated with a nonsignificant increase in the risk of major bleeding in both genders (Figure 2).

**FIGURE 3:**
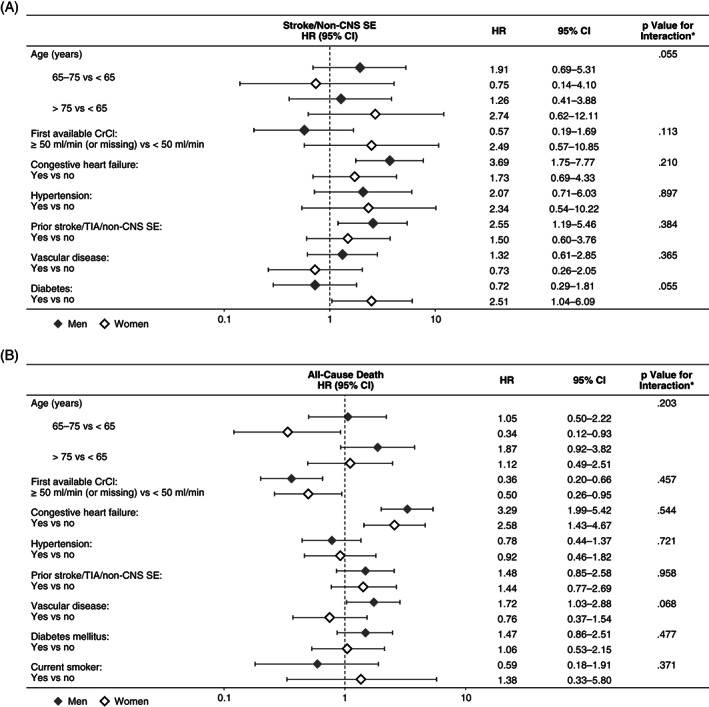
Influence of risk factors on A, stroke/nonCNS SE by gender and B, all‐cause death by gender*P value is based on the interaction between gender and the individual risk factor. CI, confidence interval; CrCl, creatinine clearance; HR, hazard ratio; nonCNS SE, noncentral nervous system systemic embolism; TIA, transient ischemic attack

No significant interactions were found between gender and risk factors for stroke/SE (Figure 3A). In women, but not men, advancing age tended to increase the risk of stroke/SE, while CHF tended to increase the risk of stroke/SE to a greater extent in men than in women. In women, there was a nonsignificant increase in the risk of stroke/SE for those with diabetes mellitus compared with those without; in contrast, in men there was a nonsignificant decrease in risk of stroke/SE for those with diabetes mellitus compared with those without diabetes.

**FIGURE 4:**
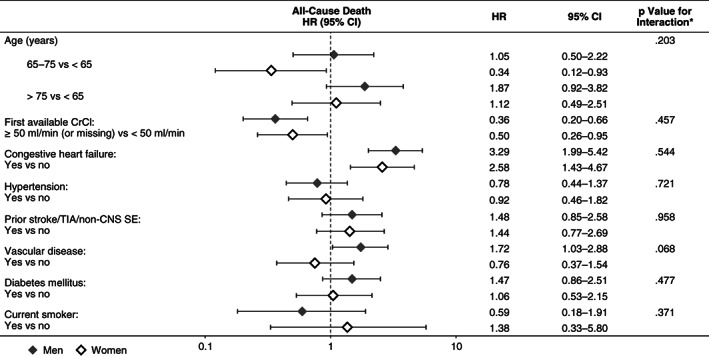
Influence of risk factors on the composite outcome of major bleeding, all‐cause death, stroke or nonCNS SE by gender *P value is based on the interaction between gender and the individual risk factor. ASA, acetylsalicylic acid; CI, confidence interval; CrCl, creatinine clearance; HR, hazard ratio; nonCNS SE, noncentral nervous system systemic embolism; NSAID, nonsteroidal anti‐inflammatory drug; TIA, transient ischemic attack

CHF was an equally important risk factor for death in men and women (Figure 3B), while the risk of death in patients with vascular disease appeared to be nonsignificantly elevated in men but not women. In addition, CHF and advancing age (> 75 years vs < 65 years) were potential risk factors for the composite outcome of major bleeding, stroke/nonCNS SE or death in both genders, while vascular disease and liver disease were associated with a nonsignificant increase in the risk of the composite outcome in men but not women (Figure 4).

## 4 Discussion

This XANTUS sub‐analysis showed that, in this cohort of rivaroxaban‐treated patients with AF, gender did not affect outcomes and the influence of different risk factors on outcomes was broadly similar between men and women.

At baseline, women were more likely than men to have a history of stroke/TIA/SE, perhaps partly due to their higher mean age. However, these rivaroxaban‐treated women were not at higher risk of stroke/SE than men, which is at odds with the inclusion of female gender as a risk factor in the CHA_2_DS_2_‐VASc scoring system.^14^ Based on the literature, however, there are contradictory data regarding the influence of female gender on stroke risk. In a recent review of 30 studies published since 1999, 17 studies identified female gender as a significant risk factor for stroke, whereas 12 of the remaining 13 studies reporting no differences in stroke risk between men and women.^15^ Meta‐analyses that predominantly included data from cohort studies have reported women to have a 1.3‐ to 2‐fold higher risk of stroke compared with men.^7^, ^16^ In our study, the similar risk of stroke/SE in both genders could also indicate greater effectiveness of rivaroxaban in women compared with men. Because all patients in the study received rivaroxaban treatment, this cannot be assessed by comparison with a control group.

Contemporary prospective registry data report disparate findings on the influence of gender on stroke outcomes; whereas data from the multinational GARFIELD AF and the US‐based ORBIT AF registries show a 1.3‐ to 1.4‐fold higher risk of stroke/SE in women compared with men after adjustments for baseline characteristics, in the European PREFER‐in‐AF registry, men and women had a similar age‐ and country‐adjusted risk of ischemic stroke/TIA/arterial embolism at 1‐year follow‐up. In all three of the aforementioned registries, anticoagulant use at baseline was similar between men and women.^10^, ^17^, ^18^ Consistent with the results of this XANTUS analysis, a meta‐analysis of phase III data from the RE‐LY, ROCKET AF, AVERROES and ARISTOTLE trials reported that, in patients receiving non‐vitamin K antagonist oral anticoagulants, the risk of stroke/SE was similar between men and women. In the same meta‐analysis, data from patients treated with vitamin K antagonists in the SPORTIF III‐IV, RE‐LY, ARISTOTLE, ROCKET AF and BAFTA trials showed that the risk of stroke/SE was higher in women than men.^19^ Interestingly, in GARFIELD AF, anticoagulation (which consisted of vitamin K antagonists in about 73% of patients treated, irrespective of gender) appeared to be less effective at reducing the risk of stroke/SE in women than in men.^10^ Together, these data suggest that gender differences in stroke risk may be more pronounced with vitamin K antagonists than non‐vitamin K antagonist oral anticoagulants. The reasons for this are unclear, but may reflect poorer vitamin K antagonist control in women compared with men ‐ indeed, female gender is included as a risk factor in the SAMe‐TT_2_R_2_ score, which can predict poor international normalized ratio control.^20^ Further research is needed to investigate whether gender differences in stroke risk may be influenced by class of oral anticoagulant.

A recent meta‐analysis of data from cohort studies reported a 12% higher risk of all‐cause mortality in women compared with men with AF.^7^ However, consistent with data from GARFIELD AF, no differences in mortality between genders were found in our study. In contrast, in ORBIT AF, after adjustment for differences in baseline characteristics, mortality was more than 40% lower in women than men. Gender is not included as a risk factor for major bleeding in the HAS‐BLED, ATRIA or ORBIT bleeding scores.^21^, ^22^, ^23^ Accordingly, data from the GARFIELD AF, ORBIT AF and PREFER‐in‐AF registries show no differences in major bleeding outcomes between women and men.^10^, ^17^, ^18^ In our study, men tended to have a higher risk of major bleeding than women, although this did not reach nominal statistical significance. Nonetheless, this is broadly consistent with post hoc analyses from ROCKET AF and ARISTOTLE trials, which report female gender to be associated with a lower risk of major bleeding in multivariate analyses.^24^, ^25^ A meta‐analysis has also shown women with AF treated with non‐vitamin K antagonist oral anticoagulants have a lower risk of major bleeding than men.^19^ Because the ages and gender distributions in the phase III trials, meta‐analyses of phase III trials and registry studies were broadly similar, it is unlikely that these characteristics contributed substantially to the different findings of the studies (Table 2).

**TABLE 2:** Summary of studies assessing the effect of gender on outcomes in patients with atrial fibrillation

Study	Population age^a^ and gender distribution	Reported effect of gender on outcome(s)^b^		
		Stroke/SE	Major bleeding	Mortality
Randomized controlled trials				
Subanalysis of risk factors for major bleeding in ROCKET AF^24^	Median age 73 years, IQR 65,78 40% female patients	NR	Lower risk in women	NR
Subanalysis of risk factors for major bleeding in ARISTOTLE^25^, ^26^	Median age 70 years, IQR 63,76 35% female patients	NR	Lower risk in women	NR
Observational studies				
Prospective GARFIELD‐AF registry study^10^	Mean age 72 ± 10.4 years in women and 68 ± 11.7 years in men 44% female patients	Higher risk in women^c^	No significant difference^c^	No significant difference^c^
Prospective ORBIT AF registry study^17^	Median age 77 (69,83) years in women and 73 (65,80) years in men 42% female patients	Higher risk in women^c^	No significant difference	Lower risk in women (all‐cause and CV death)^c^
Prospective PREFER registry study^18^	Mean age 70 ± 10.7 years in men and 74 ± 9.7 years in women 40% female patients	No significant difference^c^	No significant difference^c^	NR
Reviews and meta‐analyses				
Review of 30 observational and randomized controlled trials^15^	Overall age and gender distribution in patients with AF not reported	Higher risk in women (17 studies); no significant difference (12 studies); higher risk in men (one study)	NR	NR
Meta‐analysis of 30 cohort studies^7^	Age 45 to 83 years Overall proportion of female patients with AF not reported	Higher risk in women	NR	Higher risk in women (all‐cause and CV death)
Meta‐analysis of five randomized controlled trials and 12 prospective observational studies^9^	Mean age 62 to 83 years 28% to 53% female patients	Higher risk in women	NR	NR
Meta‐analysis of six randomized controlled trials^19^	Mean age 70 to 81 years Overall proportion of female patients not reported	No significant difference (with NOACs), or higher risk in women (with VKA therapy)	Lower risk in women (with NOACs), or no difference between genders (with VKA therapy)	NR

Abbreviations: AF, atrial fibrillation; CV, cardiovascular; IQR, interquartile range; NOAC, nonvitamin K antagonist oral anticoagulant; NR, not reported; SE, systemic embolism; TIA, transient ischemic attack; VKA, vitamin K antagonist.

^a^ Where reported, age is shown as median (IQR) or mean ± SD.

^b^ Outcome definitions may differ between studies.

c Adjusted for differences in baseline characteristics.

Our analysis also investigated the influence of different risk factors on treatment‐emergent outcomes in men and women, an approach used in the GARFIELD AF study to investigate the influence of gender on outcomes. Although not statistically significant, CHF was a potential risk factor for death in both genders, consistent with GARFIELD AF.^10^ Vascular disease tended to be more often associated with worse outcomes in men than women, while diabetes tended to increase the risk of stroke in women, consistent with observations from a cohort of patients without AF.^27^ However, trends were not statistically significant and the low event rates (particularly for the individual endpoints) resulted in wide confidence intervals, meaning our findings should be interpreted with caution.

### 4.1 Limitations

Enrolment into XANTUS was based on voluntary participation by centers and patients, which might have created patient or physician selection bias. Predefined criteria for events and central adjudication are means to ensure internal validity of the results, but replication in independent cohorts is warranted.

Lack of information is a common drawback of real‐world studies, and a limitation of this analysis is the fact that data, such as CrCl levels, were missing for some patients. Because the baseline characteristics of patients with unknown CrCl were very similar to those with CrCl >50 mL/minutes, they were included in the same group of patients. Furthermore, the low burden of cardiovascular disease in the group with missing CrCl suggests renal impairment is unlikely to be present in most of these patients. However, the missing CrCl levels in the current study add some uncertainty to the results.

Additionally, because event rates were generally low, the lack of differences in the study outcomes between men and women does not entirely exclude the possibility that there may in fact be some differences between genders in rivaroxaban‐treated patients with AF.

## 5 Conclusions

This XANTUS sub‐analysis showed that the rates of major bleeding, symptomatic thromboembolic events and death in patients with AF receiving rivaroxaban were similar in men and women; there were no significant differences in risk factors for these outcomes between genders. Further research is needed to better characterize the relative importance of different risk factors on outcomes in men vs women and to determine whether gender differences exist in patients treated with vitamin K antagonists or non‐vitamin K antagonist oral anticoagulants.

## CONFLICT OF INTEREST

A. John Camm has received institutional research grants and personal fees as an advisor or speaker from Bayer, Boehringer Ingelheim, Pfizer/BristolMyers Squibb and Daiichi Sankyo. Pierre Amarenco has served as a consultant for Bayer, BristolMyers Squibb, Pfizer, Boehringer Ingelheim, Daiichi Sankyo, AstraZeneca, Sanofi, Boston Scientific, Edwards, Lundbeck, Merck and Kowa Pharmaceutical. Sylvia Haas has served as a consultant for Aspen, Bayer, BristolMyers Squibb, Daiichi Sankyo, Pfizer, Portola and Sanofi. Miriam Bach and Silvia Kuhls are employees of Bayer AG. Paulus Kirchhof has received research support from the European Union, the British Heart Foundation (London, UK), the Leducq Foundation (Paris, France), the German Centre for Cardiovascular Research (DZHK, Berlin, Germany) and from several drug and device companies active in AF; he has also received honoraria from several such companies, including Bayer, Boehringer Ingelheim, Pfizer/BristolMyers Squibb and Daiichi Sankyo. He is listed as an inventor on two pending patents held by the University of Birmingham. Marc Lambelet is an employee of Chrestos Concept, which received funding for this analysis from Bayer AG. Alexander G.G. Turpie has been a consultant for Bayer, Janssen Pharmaceutical Research & Development LLC, Astellas, Portola and Takeda.

## References

[1] Kirchhof P , Benussi S , Kotecha D , et al. 2016 ESC Guidelines for the management of atrial fibrillation developed in collaboration with EACTS. Eur Heart J. 2016;37:2893‐2962. 27567408 10.1093/eurheartj/ehw210

[2] Chugh SS , Havmoeller R , Narayanan K , et al. Worldwide epidemiology of atrial fibrillation: a global burden of disease 2010 study. Circulation. 2014;129:837‐847. 24345399 10.1161/CIRCULATIONAHA.113.005119 PMC4151302

[3] Zoni‐Berisso M , Lercari F , Carazza T , Domenicucci S . Epidemiology of atrial fibrillation: European perspective. Clin Epidemiol. 2014;6:213‐220. 24966695 10.2147/CLEP.S47385 PMC4064952

[4] Schnabel RB , Yin X , Gona P , et al. 50 year trends in atrial fibrillation prevalence, incidence, risk factors, and mortality in the Framingham Heart Study: a cohort study. Lancet. 2015;386:154‐162. 25960110 10.1016/S0140-6736(14)61774-8 PMC4553037

[5] Feinberg WM , Blackshear JL , Laupacis A , Kronmal R , Hart RG . Prevalence, age distribution, and gender of patients with atrial fibrillation. Analysis and implications. Arch Intern Med. 1995;155:469‐473. 7864703

[6] Gillis AM . Atrial fibrillation and ventricular arrhythmias: sex differences in electrophysiology, epidemiology, clinical presentation, and clinical outcomes. Circulation. 2017;135:593‐608. 28153995 10.1161/CIRCULATIONAHA.116.025312

[7] Emdin CA , Wong CX , Hsiao AJ , et al. Atrial fibrillation as risk factor for cardiovascular disease and death in women compared with men: systematic review and meta‐analysis of cohort studies. BMJ. 2016;532:h7013. 26786546 10.1136/bmj.h7013 PMC5482349

[8] Lang C , Seyfang L , Ferrari J , et al. Austrian Stroke Registry Collaborators. Do women with atrial fibrillation experience more severe strokes? Results from the Austrian stroke unit registry. Stroke. 2017;48:778‐780. 28151397 10.1161/STROKEAHA.116.015900

[9] Camm AJ , Amarenco P , Haas S , et al. XANTUS: a real‐world, prospective, observational study of patients treated with rivaroxaban for stroke prevention in atrial fibrillation. Eur Heart J. 2016;37:1145‐1153. 26330425 10.1093/eurheartj/ehv466 PMC4823634

[10] Camm AJ , Accetta G , Al Mahmeed W , et al. Impact of gender on event rates at 1 year in patients with newly diagnosed non‐valvular atrial fibrillation: contemporary perspective from the GARFIELD‐AF registry. BMJ Open. 2017;7:e014579. 10.1136/bmjopen-2016-014579 PMC5353285 28264833

[11] Camm AJ , Amarenco P , Haas S , et al. XANTUS: rationale and design of a noninterventional study of rivaroxaban for the prevention of stroke in patients with atrial fibrillation. Vasc Health Risk Manag. 2014;10:425‐434. 25083135 10.2147/VHRM.S63298 PMC4108256

[12] Schulman S , Kearon C . Definition of major bleeding in clinical investigations of antihemostatic medicinal products in non‐surgical patients. J Thromb Haemost. 2005;3:692‐694. 15842354 10.1111/j.1538-7836.2005.01204.x

[13] Bayer AG . Xarelto® (rivaroxaban) Summary of Product Characteristics. Bayer AG: Leverkusen, Germany 2020 https://www.ema.europa.eu/documents/product-information/xarelto-epar-product-information_en.pdf.

[14] Lip GYH , Nieuwlaat R , Pisters R , Lane DA , Crijns HJGM . Refining clinical risk stratification for predicting stroke and thromboembolism in atrial fibrillation using a novel risk factor based approach: The Euro Heart Survey on Atrial Fibrillation. Chest. 2010;137:263‐272. 19762550 10.1378/chest.09-1584

[15] Cheng EY , Kong MH . Gender differences of thromboembolic events in atrial fibrillation. Am J Cardiol. 2016;117:1021‐1027. 26923085 10.1016/j.amjcard.2015.12.040

[16] Wagstaff AJ , Overvad TF , Lip GYH , Lane DA . Is female sex a risk factor for stroke and thromboembolism in patients with atrial fibrillation? A systematic review and meta‐analysis. QJM. 2014;107:955‐967. 24633256 10.1093/qjmed/hcu054

[17] Piccini JP , Simon DN , Steinberg BA , et al. Differences in clinical and functional outcomes of atrial fibrillation in women and men: two‐year results from the ORBIT‐AF registry. JAMA Cardiol. 2016;1:282‐291. 27438106 10.1001/jamacardio.2016.0529

[18] Schnabel RB , Pecen L , Ojeda FM , et al. Gender differences in clinical presentation and 1‐year outcomes in atrial fibrillation. Heart. 2017;103:1024‐1030. 28228467 10.1136/heartjnl-2016-310406 PMC5529986

[19] Pancholy SB , Sharma PS , Pancholy DS , Patel TM , Callans DJ , Marchlinski FE . Meta‐analysis of gender differences in residual stroke risk and major bleeding in patients with nonvalvular atrial fibrillation treated with oral anticoagulants. Am J Cardiol. 2014;113:485‐490. 24315113 10.1016/j.amjcard.2013.10.035

[20] Apostolakis S , Sullivan RM , Olshansky B , Lip GYH . Factors affecting quality of anticoagulation control among patients with atrial fibrillation on warfarin: the SAMe‐TT_2_R_2_ score. Chest. 2013;144:1555‐1563. 23669885 10.1378/chest.13-0054

[21] Pisters R , Lane DA , Nieuwlaat R , de Vos CB , Crijns HJGM , Lip GYH . A novel user‐friendly score (HAS‐BLED) to assess 1‐year risk of major bleeding in patients with atrial fibrillation: the Euro Heart Survey. Chest. 2010;138:1093‐1100. 20299623 10.1378/chest.10-0134

[22] Fang MC , Go AS , Chang Y , et al. A new risk scheme to predict warfarin‐associated hemorrhage: The ATRIA (Anticoagulation and Risk Factors in Atrial Fibrillation) Study. J Am Coll Cardiol. 2011;58:395‐401. 21757117 10.1016/j.jacc.2011.03.031 PMC3175766

[23] O'Brien EC , Simon DN , Thomas LE , et al. The ORBIT bleeding score: a simple bedside score to assess bleeding risk in atrial fibrillation. Eur Heart J. 2015;36:3258‐3264. 26424865 10.1093/eurheartj/ehv476 PMC4670965

[24] Goodman SG , Wojdyla DM , Piccini JP , et al. ROCKET AF Investigators. Factors associated with major bleeding events: insights from the ROCKET AF trial (rivaroxaban once‐daily oral direct factor Xa inhibition compared with vitamin K antagonism for prevention of stroke and embolism trial in atrial fibrillation). J Am Coll Cardiol. 2014;63:891‐900. 24315894 10.1016/j.jacc.2013.11.013 PMC4206565

[25] Hylek EM , Held C , Alexander JH , et al. Major bleeding in patients with atrial fibrillation receiving apixaban or warfarin: The ARISTOTLE Trial (Apixaban for reduction in stroke and other thromboembolic events in atrial fibrillation): predictors, characteristics, and clinical outcomes. J Am Coll Cardiol. 2014;63:2141‐2147. 24657685 10.1016/j.jacc.2014.02.549

[26] Hylek EM, Held C, Alexander JH, et al. Major bleeding in patients with atrial fibrillation receiving apixaban or warfarin: The ARISTOTLE Trial (Apixaban for Reduction in Stroke and Other Thromboembolic Events in Atrial Fibrillation): predictors, characteristics, and clinical outcomes. J Am Coll Cardiol. 2014;63:2141‐2147. 10.1016/j.jacc.2014.02.549 24657685

[27] Almdal T , Scharling H , Jensen JS , Vestergaard H . The independent effect of type 2 diabetes mellitus on ischemic heart disease, stroke, and death: a population‐based study of 13,000 men and women with 20 years of follow‐up. Arch Intern Med. 2004;164:1422‐1426. 15249351 10.1001/archinte.164.13.1422

